# Development and acceptability of ViviendoPositivos: A culturally tailored telenovela (soap opera) intervention to improve self-management among Latinos with HIV

**DOI:** 10.1371/journal.pone.0326930

**Published:** 2025-06-30

**Authors:** Evelyn Iriarte, Natalia Villegas, Samantha Stonbraker, Paul Cook, Maria Jose Baeza, Rosina Cianelli, Christine Toledo, Kristine M. Erlandson, Catherine Jankowski

**Affiliations:** 1 College of Nursing, University of Colorado Anschutz Medical Campus, Aurora, Colorado, United States of America; 2 School of Nursing, The University of North Carolina at Chapel Hill, Chapel Hill, North Carolina, United States of America; 3 Center for Global Health Equity, University of Michigan, Ann Arbor, Michigan, United States of America; 4 School of Nursing and Health Studies, University of Miami, Coral Gables, Florida, United States of America; 5 Christine E. Lynn College of Nursing, Florida Atlantic University, Boca Raton, Florida, United States of America; 6 School of Medicine, University of Colorado Anschutz Medical Campus, Aurora, Colorado, United States of America; University of California San Diego, UNITED STATES OF AMERICA

## Abstract

Because people with HIV are living longer, there is a growing need for effective health self-management interventions. An urgent gap in care for Latino people with HIV (LWH) is the lack of culturally adapted interventions to support their health information needs. To respond to this need, we developed *ViviendoPositivos,* a culturally tailored telenovela (soap opera) intervention to promote HIV self-management among LWH. We used a mixed methods approach to gather data for the development of the intervention from 24 Spanish or English-speaking LWHs aged ≥ 18 years. Telenovela vignettes were co-created in collaboration with a community advisory board (CAB), our research team, and a telenovela director. Through focus groups, individual interviews, and a survey, we explored the acceptability of the telenovela vignettes (*n* = 24 LWH). Participants were mostly male (88%, *n* = 21), with an average age of 49 years (*SD* = 12) and living with HIV for 16 years (*SD = *12). During the development of *ViviendoPositivos,* CAB members identified five priority content areas: mental health and social support, HIV knowledge, adherence to antiretroviral treatment, partner communication and negotiation, and healthy aging. Contextual and cultural aspects were also incorporated into the telenovela vignettes. Participants reported high satisfaction with the information and stories presented (92%, *n* = 22) and mentioned that the topics were relatable. All participants expressed willingness to watch the telenovela with a preference for online streaming via a website. The final product was an HIV self-management telenovela series of five 10-minute filmed episodes that incorporated the ideas and feedback of LWH.

## Introduction

The proportion of newly diagnosed HIV cases continues to rise among Hispanic/Latino/a/x communities (hereafter referred to as Latinos), the largest ethnic minority group in the U.S. [1,2]. Latino people with HIV (LWH) face disparities in health at many stages along the HIV care continuum. Also, it is estimated that only 65% of LWH are virally suppressed, far below the 95% national goal [3]. As advances in treatment have transformed HIV from an acute to a chronic disease and people will likely experience challenges associated with living with HIV and aging, effective HIV self-management interventions are needed for LWH [4].

Self-management is defined as an “individual’s intrinsically controlled ability to live with the medical, role, and emotional consequences of chronic diseases (e.g., HIV) in collaboration with healthcare providers and social networks” [5]. LWH need to self-manage their course of treatment, including maintaining antiretroviral therapy (ART) adherence, physical health, psychosocial functioning, and daily adaptations to living with a stigmatized chronic illness. As a result, LWH need to be prepared with self-management behaviors and skills to face the challenges of aging with HIV [4,6,7]. Improving HIV self-management can help prevent the progression of HIV, reduce hospitalization and referrals to care centers, lead to better clinical (e.g., viral suppression, CD4 counts, adherence to ART) and care results (e.g., engagement and retention in care), and reduce the incidence of comorbidities and mortality [8–10]. HIV self-management is critical for sustained HIV care, but there is limited evidence supporting culturally tailored self-management strategies for LWH [11].

An education-entertainment approach integrates educational messages into popular entertainment formats like media, offering an alternative to traditional methods of delivering educational content [12]. Education-entertainment media interventions use prosocial messages to promote health behavior change through social role modeling [13], making it a suitable medium for embedding health messages within the sociocultural context of the target audience. It has been shown that viewing others (e.g., characters) engaging in health behaviors through realistic education-entertainment narratives significantly enhances viewers’ health knowledge, attitudes, intentions, and behaviors [14,15].

Telenovelas (soap operas) are serial dramas presented on daytime radio, television, streaming services, or websites and are chiefly characterized by tangled interpersonal situations and melodramatic or sentimental treatment [16,17]. Using an education-entertainment approach, telenovelas easily serve as an innovative medium for health education through narratives that document cultural expressions and address sensitive issues related to social and situational vulnerabilities [17,18]. Telenovela interventions have been used to decrease substance use, risky sexual behaviors, and caregiver burden and to improve breast cancer screening rates, knowledge and preventive behaviors in renal disease, and mental health literacy in Latinos [17]. Despite the existence of telenovelas targeting HIV prevention among Latino communities in South Florida and California, to our knowledge, no existing telenovelas have focused on promoting HIV self-management among LWH [17,19]. This study aimed to describe the development process for co-creating a culturally tailored filmed dramatized story (telenovela/soap opera intervention) to promote self-management among LWH and assess its acceptability.

## Materials and methods

### Study design

A convergent mixed method design, which collects and interprets quantitative and qualitative data, was used in this study [20,21]. Phase 1 was a collaborative effort to develop culturally tailored telenovela stories for HIV self-management among LWH. Entitled “*ViviendoPositivos*” (LivingPositives), the stories were co-created by a 6-member community advisory board (CAB) of LWH, an expert panel (research team and consultants), and a telenovela director using vignettes to ensure a diverse range of perspectives and experiences were represented. Phase 2 aimed to explore the acceptability of the telenovela stories/vignettes among a group of LWH.

### Theoretical framework

During development, *ViviendoPositivos* incorporated social cognitive theory [13], narrative engagement theory [14], and research on emotional responses as action-guiding intervention delivery mechanisms [22]. These theories reinforce the assertion that LWH will relate to the content and narratives of the telenovela in a manner that enhances both their engagement and understanding, promoting the adoption of health behaviors. As such, *ViviendoPositivos* engages LWH in the narrative by watching the telenovela (interest) and relating to the message (realism) and the characters (identification).

Social determinants of health were portrayed in the telenovela to ensure that the content was culturally relevant for the audience. The telenovela featured scenarios depicting barriers to accessing quality HIV-related healthcare, such as financial and language barriers, lack of health insurance, and mistrust in the healthcare system [23]. To address the issue of low health literacy, the telenovela incorporated scenes where characters discuss misunderstandings about HIV and its treatment. Moreover, the telenovela stories portray the influence of social support networks, cultural values (e.g., machismo, marianismo, familism), and stigma on health behaviors. Machismo in Latino culture refers to the belief in male dominance, portraying men as authoritative and in control of their environment, including over women, especially in sexual relationships [24,25]. The counterpart, Marianismo, emphasizes female submissiveness, purity, and self-sacrifice for family harmony [26,27]. Familism highlights the importance of family loyalty and solidarity in Latino communities [28]. For instance, characters in the telenovela face stigma from family or community members due to their HIV status, affecting their willingness to seek care or disclose their status.

### Participants and setting

The study was conducted in the Denver Metropolitan Area, CO. In this location, LWH represent about 25% of the population living with HIV [29]. A convenience sample of 24 LWH from the community participated in the development of the telenovela. Participants were asked to be part of a community advisory board (CAB) to create the telenovela stories/vignettes (Phase 1, *n* = 6) and/or to evaluate the acceptability of the telenovela stories/vignettes (Phase 2, *n* = 24). Participants could be part of both phases. Inclusion criteria were: a) self-report as living with HIV, b) age ≥ 18 years, c) identify as Hispanic or Latino/a/x, d) provide written consent, e) reside in the Denver Metropolitan Area, and f) being Spanish or English-speaker.

Recruitment was conducted between September 2023 and February 2024. The research team conducted face-to-face recruitment in a community-based organization located in Denver, CO. Our community partner for this study was a statewide AIDS Service community-based organization offering care to over 5,200 clients with HIV and prevention services for those at higher risk of acquiring HIV, which facilitated this study by assisting with recruitment and providing space for study activities. In addition, flyers in Spanish and English were placed in the community (e.g., HIV clinics and other community-based organizations), and referrals from other research studies with LWH were used as recruitment strategies. The study was approved by the Institutional Review Board of the University of Colorado (#23–1129), and all subjects gave written informed consent before data collection.

### Procedures and data collection

#### Phase 1: Vignettes development.

Vignettes, short stories about hypothetical individuals presented to glean information about their beliefs, create a story that participants can relate to [30]. Vignettes can reduce discomfort and encourage sharing of personal experiences [31]. This study used vignettes to describe decisions and behaviors that LWH may exhibit in real-life scenarios. Participants were asked how the character might feel or act or what they would do, making vignettes useful for sensitive topics by allowing discussion of third-person scenarios.

First, our interdisciplinary research team worked with a CAB of LWH (*n* = 6) for story designing and development through iterative participatory group activities, where participants were asked to attend three weekly sessions. Each session was facilitated in Spanish and led by the principal investigator using a semi-structured guide. Sessions included guided brainstorming for HIV self-care management strategies, specific life experiences that might inform vignettes, ranking of relevant content, role-playing, and group discussions. CAB sessions were audio-recorded to track the process. A second team member took notes regarding details for vignettes, storylines, behaviors of the characters, and general observations of the group dynamic. At the end of the CAB sessions, participants were compensated $30 for each 90-minute session. Table 1 describes the activities for each session.

**Table 1:** Summary of the community advisory board session activities.

# Session	Aim of the session	Activities	Example of questions in the semi-structured guide
1	To define HIV self-management relevant content for LWH	The research team and the consultant elaborated on a preliminary draft with the proposed contents based on a literature review. CAB members guided the inclusion/ modification of the HIV self-management-related content and beliefs in the telenovela. A semi-structured guide with a PowerPoint presentation was used to prompt this group discussion.	What does self-management mean to you? How do you think self-management relates to HIV? What is important for you to know so that you can manage your HIV? What would you like to learn about how to manage HIV? Barriers and Facilitators Which cultural values are relevant to the stories we want to create? (e.g., familism, machismo, marianismo?)
2	To identify the structure of the telenovela stories	CAB members guided the development of the characters (i.e., personality, relationships between the characters, setting) and how to make the telenovela entertaining. This step was guided by an arrangement of PowerPoint slides with HIV self-management case scenarios developed by the research team (based on Session 1) and a semi-structured guide. After that, CAB members were instructed to create a storyline of a fictitious situation of a LWH character and the HIV self-management issues that he/she would face to manage this situation.	If a telenovela is available for HIV self-management, what would you like to see in it? For example, what type of stories? Can you think of specific situations you would like to see in the telenovela? For example, has something happened to you that you think would be helpful for others to know about? Please tell me what you think about the characteristics of the telenovela in terms of length, characters, settings, actors and roles in the telenovela, use of humor/style of the telenovela (e.g., drama, comedy, etc.) What do you think about including characters from different nationalities or accents in the stories?
3	To design the vignette storyline	CAB members created and shared their own stories (vignettes) and role-played the stories they composed. This was followed by a semi-structured group discussion reflecting on the play.	What do you think about the storylines you created? What would you like to modify?

CAB = community advisory board; HIV = Human immunodeficiency virus; LWH = Latino people with HIV.

After conducting the CAB sessions, the CAB members and the research team decided which stories seemed more appealing and engaging to the target audience. These stories were reviewed during two online weekly meetings (90 minutes each), and transcripts and field notes from the CAB sessions were reviewed. Once the core themes and stories for the telenovela were established, a Latino bilingual theater director and screenwriter, with prior experience developing and filming telenovelas for Latino people, were brought in to write and refine the final vignettes, enhance the dramatic elements, and ensure cultural authenticity while maintaining the integrity of the elements and stories suggested by the CAB. This sequence ensured that the intervention was both community-driven and professionally produced. Then, these materials were reviewed by three research team members for final approval.

#### Phase 2: Acceptability of the telenovela vignettes.

Through focus groups (*n* = 4) and individual interviews (*n* = 12), we explored the acceptability of the telenovela vignettes with 24 LWH. We used both data collection methods so that we could still ascertain the perspectives and opinions of individuals uncomfortable with group activities while also fostering community, support, and interactive dialogue among those willing to share their experiences in a group setting. This dual approach ensured all participants could provide input in a way that respected their privacy and preferences. The final sample size was determined using the concept of saturation (when no new data emerges during data collection) [32]. Each 60-minute focus group was conducted in Spanish. Individual interviews were conducted in person in a private room located in the community-based organization, in English or Spanish, according to participant preference, and lasted 20–30 minutes. During the focus groups and individual interviews, participants reviewed the co-created vignettes, which were presented as narrated script videos. As the telenovela had not yet been filmed, this format allowed participants to provide feedback on the storylines and structure prior to final production. To evaluate the acceptability of the content, participants shared their opinions on the telenovela’s themes, characters, and overall structure. A bilingual team member led data collection using a semi-structured guide. Examples of the questions in the guide included:

- *What do you think about the telenovela for* HIV self-management *that you watched? What aspects did you like or dislike, and why?*
- *What were some of the good things about this telenovela? Do you think the telenovela provided good or useful information? How would you use this information to improve your health?*
- *What aspects would you modify? And why? What other content should we add to the telenovela? Is there anything missing?*
- *Please tell me what you think about the characteristics of the telenovela in terms of length, characters, setting, and use of humor/style of the telenovela (e.g., drama, comedy, etc.)*

Participants also completed a survey that included a socio-demographic and health status form (e.g., age, country of origin, years living in the U.S., etc.) and questions about the acceptability of content in REDCap. We assessed acceptability by asking participants to respond to a 10-item acceptability questionnaire [19]. Participants were compensated $30 for completion of the interview or focus group and survey.

### Data analysis

NVivo Artificial Intelligence-based transcription software transcribed all the audio recordings verbatim in Spanish and English. The transcriptions were reviewed to address any discrepancies with the audio and delete identifying information. For phase 1, conventional content analysis was used to identify the major themes related to the development of the telenovela vignettes [33]. In this method, coding categories are derived directly from the text data using an inductive method to identify recurring content [33]. Data analysis was conducted using NVivo 14, and the themes and codes were built by analyzing the data line by line and then grouping the codes into clusters to create themes [34]. To conduct this process, two bilingual in English and Spanish research team members independently read through the interview transcripts [33]. Transcripts were coded in their original language to preserve participants’ wording and meaning. The two bilingual research team members independently coded transcripts and met every three transcripts to discuss and reach a consensus. New codes were added as needed. Final codes were categorized by mutual agreement to keep qualitative rigor [21]. Final results and quotations from Spanish transcripts were translated into English for publication using a back translation process. Three research team members reviewed and modified the vignettes and scripts according to the feedback from the focus groups/interviews and the survey.

To ensure rigor [21], dependability was supported through detailed documentation of the analysis process, including raw data such as direct quotes from transcripts, a codebook, and a code sheet. Credibility was strengthened by having two researchers independently code the transcripts and by incorporating verbatim quotes to accurately capture participants’ experiences. All authors were deeply engaged with the study procedures, topics, and qualitative methodologies. The research team, comprising bilingual Latina researchers with expertise in HIV intervention development, brought valuable cultural and contextual insight to the analysis. Prior collaborations with the community helped foster trust during recruitment and data collection, and the team engaged in ongoing reflexive practice to consider how their positionality may have shaped the interpretation of findings. To enhance transferability, participant demographics and study context were thoroughly described to support replication with Latino communities in other settings [21].

For Phase 2, an integration analysis was conducted. We analyzed the quantitative and qualitative data separately, locating integration in the design through a joint display (table) and drawing meta-inferences or insights from it. Quantitative analyses were performed to calculate descriptive statistics using IBM SPSS Statistics. The bilingual research team analyzed qualitative data in their original language through conventional content analysis, similar to Phase 1 [20]. Lastly, quantitative and qualitative data were merged and interpreted [35,36].

## Results

### Phase 1: Vignettes development

The mean age for the CAB members (*n* = 6) was 48 years old (*SD* = 17, range: 28–67). All participants were men who have sex with men, with an average of 11 years of education (*SD* = 6, range: 3–16). Most participants were from Mexico (33%); their preferred language for daily life was Spanish (83%). The mean time since HIV diagnosis was 16 years (*SD* = 14, range: 0.5–32); 83% of the participants were on ART.

Through the vignette development process, three key themes emerged: 1) Conceptualization of self-management for Latinos with HIV, highlighting how participants define and navigate self-management in the context of HIV; 2) Content for the telenovela, identifying priority topics such as mental health, adherence to ART, and partner communication; and 3) Telenovela-specific features, describing participants’ preferences for storyline structure, character development, and cultural representation.

#### Theme 1: Conceptualization of self-management for Latinos with HIV.

CAB members reported that self-management in the context of HIV includes independence and liberty to manage HIV. They referred to the importance of managing HIV for overall well-being and health. One participant noted: *“Self-management is to take care of my health and take all my medications. When I was diagnosed with HIV, I realized that my health was very important”* [male, 43 years old]. Another participant who was not on antiretroviral therapy at the time said, “*For me, it is discipline. Something I am working on because this situation happened to me [not taking the medicines] because I did not have self-control, and I did not know how to handle the situation*” [male, 28 years old]. Other participants referred to the importance of treatment and preventive behaviors to protect not only themselves but also their partners as they mentioned, “*I think that also caring for other people […] Take care of those people when they have sexual relationships or some promiscuity or a partner. Therefore, take care of the person you are with at the moment”* [male, 53 years old].

Most participants also expressed that to accomplish HIV self-management goals, it was necessary to be informed and updated about the advances in HIV prevention and care. One participant said: “*It is like a window of knowledge. The knowledge of what is new [is important], because every day there is something new in medicine. For example, right now the last thing I heard is that there will be an injection done every three months [...]. So, that could be self-management: to see if I can make a decision based on the new advances in relation to HIV”* [male, 67 years old].

#### Theme 2: Content for the telenovela.

CAB members identified five priority content areas: mental health and social support, HIV knowledge, adherence to HIV treatment, partner communication and negotiation, and healthy lifestyles in the context of aging.

Mental Health and Social Support: Mental health was the most important priority for all the participants. “*Mental health is the most important thing for me. It is the backbone* [*of* HIV self-management]” [male, 43 years old]. CAB members identified mental health issues that were important to address in the telenovela, such as internalized stigma, fear of dying when they were diagnosed, sharing the diagnosis with others to get support, and coping strategies. Taking antiretroviral therapy is a constant reminder of the burden of the disease in their lives, which impacts directly on mental health. Trauma related to living with HIV is present every time they take ART. One participant said: “*Trauma is there. Every night at nine* [this is the time he takes his HIV medication]*, I get the trauma, the usual nightmare*” [male, 67 years old].

Other contextual situations related to living with HIV also may impact mental health and, therefore, self-management: “*I live in a shelter, and the truth is that I see that many people in the situation of not having a stable home, depression eats them, and they abandon treatment*” [male, 43 years old]. Social support from family, friends, and communities was stated as relevant to overcoming the trauma that results from living with HIV. According to the participants, the telenovela stories are suggested as a platform to show the social resources available in the community. One CAB member expressed: “*But also divulge where you can find them [social resources]. One thing is that they exist [the social resources], and another thing is that people know where to go”* [male, 67 years old].

HIV-knowledge: In conjunction with the proposed topics prepared for the CAB sessions, participants identified the limited knowledge about HIV among the Latino community. They noted that the telenovela must address conceptual definitions, such as the difference between HIV and AIDS, as key to combating the lack of knowledge and ignorance. One participant noted: “*Even something as simple as people knowing the difference between HIV and AIDS*” [male, 30 years old]. Other CAB members suggested presenting situations that clearly explain concepts such as CD4 cell count, viral load, undetectable = untransmissible (U = U), and treatment as prevention. One participant noted: “*We definitely have to talk [in the telenovela] about treatment to prevent or what the word undetectable is and what the word means*” [male, 27 years old].

HIV prevention is stated as an important part of HIV self-management. Addressing HIV transmission, the use of condoms, pre-exposure prophylaxis (PrEP) use, and testing were considered key to reducing risky behaviors and promoting HIV prevention and care, as an important part of the telenovela stories. *“Since AIDS began, the use of condoms is to prevent AIDS/HIV or whatever, but many people do not understand that condoms are to prevent other diseases too”* [male, 67 years old]. CAB members also shared that it would be important to present information about PrEP to prevent HIV in the context of having a partner without HIV. They referred to a lack of knowledge about this medicine to prevent HIV: “*Prevention does exist [...], but people do not know that HIV prevention exists: PrEP treatment”* [male, 43 years old].

Adherence to ART: CAB members also shared that they would like to get information about ART adherence, as they think treatment can support self-management and prevention efforts. One participant clearly stated, “*If I take care of myself, if adhere closely to my [ART] treatment, and follow it as it is, I am taking care of others*” [male, 43 years old]. Participants also discuss the need to show how being adherent can help them achieve their therapeutic goal of being undetectable = untransmissible and the relevance that has not only for their own health but also to others’ health.

Partner Communication and Negotiation: CAB members identified sharing HIV status as the most relevant challenge to partner communication and negotiation about HIV sexual preventive behaviors. They said they do not have sufficient skills to talk about the diagnosis and negotiation of a safe sex life with their partners. *“It is not easy; it is not easy. And when you love or you are interested in the person, even more”* [male, 53 years old]. They also mentioned that fear of rejection prevents them from having open conversations with partners and that the telenovela will be a resource to support open conversations about the topic.

Cultural factors such as machismo and marianismo and the presence of intimate partner violence are relevant barriers to having open conversations and engaging in safe sex behaviors. Infidelity and the lack of HIV testing, as a partner’s behaviors that put the other at risk for HIV, were referred to as factors that put women at risk for HIV. One participant recalled that women with HIV talked about these experiences in a support group: *“I went to a support group where there were people with HIV, and I was surprised that there were many women, about seven women. So, they touched on that issue where they wanted to encourage it to be mandatory* [the HIV test] *because women, even though they were heterosexual, acquired HIV because their husbands had double lives”* [male, 30 years old].

Healthy Aging: Participants expressed interest in learning how to manage aging while living with HIV. They mentioned that the telenovela could be a platform to show those experiences (e.g., comorbidities management, polypharmacy, exercising, diet) and how to grow their self-confidence and skills for better self-management. *“What we are really looking for here is to have the image, the telenovela, of a person who lives with HIV and how that person can demonstrate their behavior and management [living with HIV and] with some other diseases that are normal of old age”* [male, 67 years old].

#### Theme 3: Telenovela-specific features.

Participants mentioned several characteristics that they wanted to incorporate into the telenovela. They preferred professional actors for the roles, and the characters should have different nationalities but not be specified from what country. *“If there is a person with HIV, it is not needed to determine from what country but rather determines that he is Hispanic”* [male, 67 years old]. Participants also said that the telenovela characters should be not only people from the lesbian, gay, bisexual, transgender, and queer or questioning (LGBTQ+) community, as most participants think that the telenovela should include all of the Latino community. “*Not just for people with HIV, but for all people in general* […] *So, it is important that the telenovela is directed to the entire Latino community, not just to the gay or LGBTQ+ communities*” [male, 67 years old].

Participants preferred stories that mix drama and comedy, and that look real to the audience. One participant said: “*Drama, but with touches of comedy*” [male, 67 years old]. Regarding realism and identification, another participant mentioned: “*I think it is real life because in real life, people scream, people cry*” [male, 43 years old]. Participants also said that the telenovela episodes should be short to avoid boredom. One participant said: “*Ten minutes is fine because people do not get bored, and ten minutes goes by quickly”* [male, 53 years old].

#### Telenovela vignettes.

Based on the previous findings, the CAB, the director and scriptwriter, and the research team co-created six vignettes. These vignettes covered a range of HIV self-management topics (HIV knowledge, adherence to ART, mental health, social support, healthy aging, and partner communication). After reviewing the vignettes with the research team and the telenovela director, we consolidated them into five final episodes. This decision was based on thematic overlap and narrative flow, ensuring that each episode remained engaging while comprehensively addressing key self-management concepts. Specifically, the vignettes on healthy aging and partner communication and negotiation were merged into one episode to streamline content while preserving the core messages. The final five episodes maintained the original participant-driven themes while enhancing coherence and storytelling effectiveness. Table 2 describes each episode, vignettes, key contents, and cultural aspects covered in each episode.

**Table 2:** Episodes, vignettes, key content, and cultural aspects covered in the telenovela intervention.

Episode	Vignette	Key content	Cultural aspects
**Episode 1:** **Living with HIV** Topic: HIV knowledge	María, 35, has been married to Felipe for ten years and living with HIV for five. After discovering Felipe’s infidelity and abusive behavior due to his drinking, María gets tested for HIV and finds out that she acquired the virus from him. Initially in denial, she seeks help from a social worker, who advises her to get HIV treatment. María faces challenges with insurance and finding a doctor who speaks her language, but she eventually starts treatment after receiving assistance.	Definition of HIV/AIDS, transmission and prevention Immune system explanation CD4 cells and viral load Chronicity of HIV Access to care barriers: insurance, language, lack of education Treatment to prevent (U = U) and initiation of treatment	Gender roles (machismo and marianismo) and HIV risk HIV stigma Language and access barriers Strong sense of community within Latino culture
**Episode 2:** **A second opportunity** Topic: Adherence to HIV treatment	Juan, 29, learns he acquired HIV after an unprotected encounter with Pedro, who responds harshly when confronted. Depressed and overwhelmed, Juan delays treatment for months. After speaking with a supportive friend, he visits a doctor who advises him to start ART and explains the importance of adherence. Juan begins treatment and shares his experience with a support group.	Mental health barrier to adherence Social support Consequences of not being adherent: increased risk of transmission, AIDS Treatment as prevention (U = U) and initiation of treatment	Gender roles (machismo) and HIV risk Stigma (HIV, mental health, sexual orientation) Familism Respect for healthcare providers Strong sense of community within Latino culture
**Episode 3:** **Sofía as a president: A journey to empowerment** Topic: Mental health and social support	Sofia, 40, has lived with HIV for ten years but kept it secret due to fear of rejection. In the support group, she shares that she acquired HIV from an unprotected encounter on a dating app, which happened after she had been drinking alcohol and felt less in control of her decisions. With help from a community organization, Sofía receives psychological support and connects with local resources. She eventually confides in her friend Nora, who supports her, and together, they participate in HIV prevention activities in their community.	Importance of social support HIV stigma HIV testing Substance use (alcohol) Condom use	Marianismo Stigma (HIV and mental health) Strong sense of community within Latino culture
**Episode 4: Healthy aging**^**a**^ Topic: Healthy lifestyles in the context of aging	Don José, 60, has lived with HIV for over 30 years and was recently diagnosed with diabetes and hypertension. Worried about managing his health, he joins a gym after his doctor advises exercise and a healthy diet. There, he starts a romantic relationship with a woman who refuses to use a condom, believing she cannot get pregnant. Aware of his status as U = U, Don José insists on condom use and discusses PrEP with her, receiving support from his daughter Carla.	Healthy behaviors: diet, exercise, medications, etc. Mental health management Management of comorbidities Social support	Familism Strong sense of community within Latino culture Stigma (HIV)
**Episode 5:** **I take care of myself and prevent**^**a**^ Topic: Partner communication and negotiation	Matías, 30, has been living with HIV for 5 years and fears telling his partner, Patrick, 25. Matías shares his HIV status on his birthday, and Patrick initially reacts angrily. After some days, Patrick returns to talk, and Matías explains HIV preventive behaviors such as U = U, condom use, and PrEP. Matías then invites Patrick to join him at a doctor’s appointment, where they both learn how to maintain a safe sexual relationship.	Treatment as prevention (U = U) Communication and negotiation with the couple (addressing fear of rejection, managing confrontation) Sharing HIV diagnosis Condom use (to prevent not only HIV but STDs) HIV testing Knowledge, access, and use of PrEP	Gender roles and relationship dynamics Respect for healthcare providers Strong sense of community within Latino culture

^a^There is an overlap in the filmed version of these episodes.

AIDS = Acquired immunodeficiency syndrome; HIV = Human immunodeficiency virus; PrEP = pre-exposure prophylaxis; STDs = sexually transmitted diseases; U = U = undetectable = untransmissible.

### Phase 2: Acceptability of the telenovela vignettes

Of the 24 participants in phase 2, the mean age was 49 years old; participants were primarily male, men who have sex with men as the primary HIV risk factor, with an average of 13 years of education. The majority of the participants were born in Mexico and the U.S.; their preferred language for daily life was Spanish. The mean time since HIV diagnosis was 16 years; 96% of the participants were on ART. A detailed description of the characteristics of the participants in Phase 2 can be found in Table 3.

**Table 3:** Participant characteristics phase 2 (*n* = 24).

Sociodemographic Characteristics	*M (SD)*	*n (%)*
Age (years) (range: 28–67)	49.2 (12.2)	
Education (years)^a^ (range: 2–24)	13.2 (5.3)	
Monthly per capita income (U.S. dollars)^a^ (range: 0–5000)	1848.7 (1332.1)	
Country of origin		
Mexico		11 (45.8)
U.S.		4 (16.7)
Puerto Rico		3 (12.5)
Venezuela		2 (8.3)
Other^b^		4 (16.7)
Years living in the U.S.^c^ (range: 1–44)	15.7 (11.8)	
Preferred language (Spanish)		18 (75.0)
Race		
Mixed race		14 (58.3)
White		10 (41.7)
Marital status		
Single		17 (70.8)
In a relationship, not legally married		4 (16.7)
Married		1 (4.2)
Divorced/separated		1 (4.2)
Widowed		1 (4.2)
Religion		
Catholic		14 (58.3)
Other		4 (16.7)
None		6 (25.0)
Current employment status (yes)		16 (66.7)
Gender identity		
Male		21 (87.5)
Female		2 (8.3)
Trans (female)		1 (4.2)
Sexual orientation		
Homosexual/Gay		17 (70.8)
Heterosexual		5 (20.8)
Bisexual		1 (4.2)
Asexual		1 (4.2)
Health Characteristics		
Years living with HIV infection^a^ (range: 0.5–40)	16.2 (12.1)	
Health insurance (insured)		21 (87.5)
Comorbidities (yes)		14 (58.3)
On ART (yes)		23 (95.8)

ART = antiretroviral treatment for HIV; *M* = mean; *N* = sample size; *SD* = standard deviation; U.S.: United States; % = percentage.

^a^Missing data for one case.

^b^Colombia, Salvador, Nicaragua, Honduras.

^c^Years living in the U.S. were calculated by excluding the four participants born in the U.S.

After converging qualitative and quantitative data, three key themes emerged: 1) Satisfaction with the telenovela stories, which highlighted participants’ positive feedback on the format, storylines, and content; 2) Perceived utility of the telenovela, focused on the participants’ view of the telenovela as a valuable tool for promoting holistic HIV management and educating about HIV prevention; and 3) Suggestions for improving the telenovela stories, focused on participants’ recommendations for modifying the storyline, reinforcing certain content areas, and diversifying the target audience. See Supplemental Table 1 in S1 File for joint display. These themes guided the development of the final five telenovela episodes. Participants’ preferences for watching telenovelas are summarized in Table 4.

**Table 4:** Participants’ preferences for watching telenovelas.

Telenovela preference	*n* (%)
In the last month, how often did you watch telenovelas?	
Every day	3 (12.5)
More than once a week	4 (16.7)
Once a week	1 (4.2)
Once a month	6 (25.5)
I do not watch telenovelas	10 (41.7)
At what time of the day do you watch telenovelas? (Check all that apply)	
AM	3 (12.5)
PM	21 (87.5)
If a telenovela about HIV self-management is available, how likely would you watch it?	
Completely likely	15 (62.5)
Very likely	5 (20.8)
Moderately likely	3 (12.5)
Slightly likely	1 (4.2)
Not at all likely	0 (0.0)
Where would you like to access the telenovela? (select all that apply)	
Website/Internet	14 (58.3)
Streaming services	9 (37.5)
Apps	8 (33.3)
TV	4 (16.7)
DVD	3 (12.5)
VHS	1 (4.2)
What device(s) do you use to access the internet for health information? (check all that apply)	
Cell phone	19 (79.2)
Laptop computer	8 (33.3)
Desktop computer	8 (33.3)
Tablet (iPad, Galaxy, Fire tablet etc.)	6 (25.0)
TV	4 (16.7)

#### Theme 1: Satisfaction with the telenovela stories.

Quantitative results related to the acceptability of the telenovela can be found in Table 5. During the development of *ViviendoPositivos*, participants reported high satisfaction with the information and stories presented (92%, *n* = 22). Seventy-one percent of the participants rated the telenovela as very good (*n* = 17) and 29% as good (*n* = 7). Participants’ satisfaction with the telenovela was consistent with three main elements of the telenovela: format, stories, and content.

**Table 5:** Acceptability of the telenovela.

Questions	*n* (%)
**Design**	
Rate the telenovela	
Very good	17 (70.8)
Good	7 (29.2)
Bad	0 (0.0)
Very bad	0 (0.0)
**Satisfaction with the contents and activities**	
Satisfaction with the information provided	
Satisfied/Very satisfied	22 (91.7)
Neutral	2 (8.3)
Dissatisfied/Very dissatisfied	0 (0)
Satisfaction with the stories	
Satisfied/Very satisfied	22 (91.7)
Neutral	1 (4.2)
Dissatisfied/Very dissatisfied	1 (4.2)
**Clarity and comfort**	
Understanding of the information delivered	
High/Very high	19 (79.1)
Neutral	4 (16.7)
Low/Very low	1 (4.2)
Degree of comfort with the examples used	
High/Very high	21 (87.5)
Neutral	2 (8.3)
Low/Very low	1 (4.2)
Inclusion of topics that are relevant for people like them	
Yes	21 (87.5)
Neutral	3 (12.5)
No	0 (0.0)
**Expectations**	
Fulfillment of initial expectations	
Yes	24 (87.5)
Partially	3 (12.5)
No	0 (0.0)
If we offer free access to the HIV self-management telenovela, would you like to watch it?	
Yes	24 (100.0)
Neutral	0 (0)
No	0 (0)
Likely to recommend the intervention to friends	
Yes	23 (95.8)
No	1 (4.2)
Acceptability of a potential online version	
Yes	24 (100.0)

Format: Participants mentioned that the telenovela was proficiently conducted; several referred to it as *“This is very well done.*” [Interview 001, male, 62 years old], and *“I think it is going really great. I’m pretty impressed”* [Interview 003, female, 47 years old]. Using a telenovela format helped deliver the content in an engaging form, making it more appealing for learning. Participants pointed out that traditional educational information about HIV is often perceived as heavy and unattractive. For example, one participant mentioned that the telenovela format felt more organic and natural, making the content easier to understand:

*“I liked the kind of vibe that it [the telenovela] uses; it is not a grey and sad vibe like the videos about HIV usually are. It’s a warm, normal atmosphere. I liked that, and I liked the cases, which are very realistic. I like how it [the telenovela] introduces the necessary information in a very intelligent way. It doesn’t seem forced- like if they were pushing to give me this information”* [FG1, Speaker 4, male, 30 years].

Stories: Various participants in the interviews and focus groups mentioned how the stories featured in the telenovela were appealingly realistic and portrayed real experiences and challenges of those living with HIV. This made the stories more relatable to them. *“I liked it because that [the problems faced by the characters] happen every day among people who live with HIV.”* [Interview 022, male, 50 years old]. Participants also emphasized the honest and realistic setting of the story, taking place in a support group: *“It is a [HIV] support group that meets periodically with each other, right? There are people who come in and out of the group. To me, the environment seems adequate*” [FG1, Speaker 2, male, 34 years]. The setting also effectively encouraged people to join support groups “*that [support group] invites people with HIV to participate in this type of support groups”* [FG1, Speaker 4, male, 30 years].

Content: Participants mentioned that the content was detailed, clear, and informative about HIV. One participant said: *“You explained it in detail about what happens when you become HIV positive. And you explained, if you are undetectable, you really went into detail explaining all of it”* [Interview 002, male, 54 years old]. Another participant provided a clear synthesis of the content, reflecting the clarity of the information provided: *“The first video is about how accepting when you have [HIV] or how people tolerate talking about HIV. And the second video goes deeper, talking about CD4 cells and PrEP. That is already more direct information for those who are sick and who know nothing about the disease. It is very good”* [Interview 003, female, 47 years old].

#### Theme 2: Perceived utility of the telenovela.

Most participants (88%, *n* = 21) reported that the telenovela covered topics and issues they believed were important to them. The focus group discussion and interviews reinforced this finding, suggesting that the telenovela content and activities were congruent with the participants’ needs and concerns regarding promoting holistic management of HIV and educating about HIV prevention.

Promote a holistic management of HIV: Participants mentioned that the telenovela supports HIV self-management in terms of physical (medical management, adherence to ART), psychological (mental health), and social (stigma, sharing the diagnosis with significant others or family, and social support) health. One participant mentioned that by promoting self-management, the telenovela also helps people understand that it is possible to live a healthy life with HIV:

*“[The telenovela] will help them [the watchers] accomplish to get the medication and to see their doctor on a normal basis so that they can take care of their health. They can get to story number five and realize that they can live that fruitful life and still be happy and know that they still have something, but they could still have health if they take care of themselves in the proper manner to treat their body”* [Interview 003, female, 47 years old].

Participants also noted how the telenovela can help them improve their mental health and find answers to manage the emotional toll of being diagnosed with HIV. One participant commented: “*The mental, emotional part, which I think is very important, especially when you have a lot on you, whether it be depression, whatever, […] So, I think that might help somebody get some help with medication or, how I say, mental-emotional support*” [Interview 002, male, 54 years old]. Another participant explained the benefit of the telenovela for mental health: “*Beyond physical health and physical care, I also believe in mental health because a person suffers more when they are misinformed and do not know how to handle the situations they are going to be exposed to*” [FG 3, Speaker 4, male, 43 years old]. Those who were already practicing self-care behaviors, found the telenovela to be beneficial in motivating them to keep practicing good self-management: “*Well, right now I feel very healthy, so it would help me to motivate myself or to get ahead in life”* [Interview 016, male, 47 years old].

Educate regarding HIV prevention: Participants also mentioned that the telenovela promotes HIV prevention and acceptance, highlighting that HIV can affect anyone. According to some participants, the telenovela is relevant in educating those unfamiliar with HIV and for individuals in close contact with people living with HIV. For example, one participant stated: “*I like it because, once again, it is very focused on the topic of prevention. So, I see it as positive. Not because a diagnosis is positive, but because if I have my diagnosis, I can take care of myself and inform my family, my friends, and others to prevent them from getting it. This way, they will also live positively by preventing the situation and maintaining harmony. So, it seems good to me”* [Interview 021, male, 27 years old].

By educating viewers about HIV, the telenovela also helps combat HIV stigma and dispel myths about the virus. “*Yes, that [the telenovela] would help you a lot because you would have a better understanding of what HIV really is. Many people think that by touching you, talking to you, or touching your things, they can become infected”* [Interview 019, male, 50 years old].

#### Theme 3: Suggestions to improve the telenovela stories.

All participants (100%) expressed willingness to watch the telenovela, with a preference for online streaming via a website. Ninety-six percent of the participants would likely recommend the telenovela (*n* = 23). Despite most participants saying that they would not modify any aspects of the telenovela stories, aspects to be improved included: Modifications in the storyline, reinforcement of certain contents, and diversifying the target audience.

Modifications in the storyline: These included suggestions to incorporate more content about medications and the clinical management of HIV, prioritizing the sharing of experiences among participants to delve deeper into each participant’s story, and including a greater diversity of participant stories to represent a wider range of cases and themes. For example, one participant proposed the inclusion of a social worker or interpreter: *“[…] someone who helps and guides you to find those resources. Unfortunately, I believe that our Latino community is unaware of the wealth of resources available in the community to support people with HIV, particularly Spanish speakers. These resources are truly there to support us”* [FG1, Speaker 2, male 34 years].

Other suggestions included featuring a participant who was rejected after disclosing their HIV status, someone receiving their diagnosis for the first time, a transgender participant, a physician or someone to explain medical questions, a counselor, and a couple where both partners live with HIV. Two participants also noted the telenovela nature of the intervention and suggested including more humor in the stories: *“In that story, a little more humor*” [Interview 002, male, 54 years old].

Content reinforcement: Participants also suggested strengthening specific content areas, such as the importance and significance of U = U. One participant stated: *“[Reinforce] U=U and that people learn that because I think that is the issue*” [FG 3, Speaker 4, male, 43 years old]. Including content on preventive methods was also mentioned several times, such as the use of PrEP or condoms. One participant noted, *“I think the information is very explicit, just that detail [...] there are many people out there who are having sex without a condom without knowing that PrEP exists”* [Interview 020, male, 48 years old]. Additionally, three participants suggested highlighting the most important messages at the end of each episode: “*Key messages that stand out at the end of the episode”* [FG2, Speaker 1, male, 28 years old].

Diversifying target audience: Several participants emphasized the importance of developing strategies to diversify the target audience. Participants suggested as target audience Latinos from various nationalities, individuals with different levels of disabilities, and people who acquired HIV through different ways. Overall, participants stressed the need to make the content inclusive for all segments of the Latino community. One participant reported:

*“Cultural diversity, a lot of cultural diversity so that it [the telenovela] can also be a little more empathetic with people, right? […] There can be two people who speak Spanglish maybe, or that are Mexican American, or that suddenly there are people from two completely different countries. Or […] subtitles because someone has a hard time speaking, right?”* [Interview 010, transgender woman, 33 years old]. Subtitles in English and Spanish were suggested as a way to improve accessibility to health information and to increase inclusivity.

### ViviendoPositivos telenovela intervention

Drawing on our study findings, including both full and partial convergences, and in collaboration with CAB members, the Latino community, researchers, and the director/scriptwriter, we co-created and refined *ViviendoPositivos*. The telenovela consisted of five 10-minute episodes that describe the experiences of different people living with HIV with a Latino background (men/women, older/younger, LGBTQ+ communities) who meet weekly in a social support group to talk about how they manage and live with HIV. Each episode includes an introduction, a 7–8-minute storyline, and a closing segment that highlights the key takeaways. Episodes were filmed with professional actors in Spanish with subtitles in English and Spanish. *ViviendoPositivos* is a telenovela intervention co-created by and for LWH.

## Discussion

The development and evaluation of *ViviendoPositivos* addresses a critical gap in the care continuum for LWH. Given the increasing lifespan of individuals with HIV and the health disparities that Latinos experience, there is an urgent need for culturally relevant interventions that support self-management and overall well-being. Our study provides valuable insights into the acceptability and potential impact of a novel approach to health education tailored specifically for LWH.

### Cultural tailoring and community engagement

One of the key strengths of *ViviendoPositivos* is its foundation in cultural tailoring and community engagement. By involving a CAB composed of LWH from the community, we ensured that the intervention was not only relevant but also resonated with the target audience. The identification of five priority content areas—mental health and social support, HIV knowledge, adherence to HIV treatment, partner communication and negotiation, and healthy aging—underscores the holistic approach required to address the multifaceted challenges faced by LWH.

The incorporation of contextual and cultural aspects into the telenovela vignettes and final episodes further enhanced the content’s relevance and relatability, which is crucial for fostering engagement and behavior change [17,19]. Cultural values, including machismo, marianismo, and familism, highlight how these factors influence health behaviors among LWH [11,37,38]. These cultural aspects can act as barriers to open conversations about HIV, partner communication, and engagement in safe sex practices [11,37,38]. As shown in the telenovela episodes, machismo and marianismo contribute to challenges in discussing HIV status and negotiating condom use, which can increase the risk of transmission and hinder HIV prevention. Additionally, infidelity and the lack of HIV testing by partners, especially among heterosexual women, were identified as key risk factors related to cultural norms. One participant shared an experience from a support group, noting that many women contracted HIV due to their husbands’ double lives.

These references to gender roles and cultural values, such as familism, underscore how migration and acculturation impact healthcare utilization [39]. Familism, or the emphasis on family loyalty, may complicate discussions around HIV disclosure, as individuals might fear stigmatization from family members. Addressing these cultural barriers is essential to creating effective HIV self-management interventions that resonate with the lived experiences of LWH. The culturally tailored content and stories developed by and for LWH in *ViviendoPositivos*, featuring characters facing similar situations and familiar cultural values, may serve to depict decisions that promote HIV self-management, presenting a relatable socio-cultural environment for LWH [17].

### Acceptability of ViviendoPositivos

The high levels of satisfaction reported by ninety-two percent of participants and their willingness to engage with the telenovela series reflect the intervention’s acceptability and potential for scalability. This finding is particularly significant given the historical challenges associated with engaging Latino populations in health interventions [11]. The cultural tailoring and use of a popular and culturally standard format of telenovelas to deliver critical health information can explain this finding [12,17]. The use of storytelling and dramatization not only captures the attention of the audience but also enhances the memorability and emotional impact of the messages conveyed [13,14,22]. The telenovela delivery of HIV self-management information is supported by narrative engagement theory, which posits that engaging narratives can lead to deeper cognitive and emotional involvement, ultimately influencing attitudes and behaviors [14,22]. Additionally, the preference for online streaming (100%) highlights the importance of accessibility and convenience, suggesting that digital platforms could play a vital role in disseminating health information to LWH across diverse geographic regions.

### Promoting holistic HIV self-management

Participants in our study emphasized the importance of including basic HIV information, with a particular focus on promoting mental health. This study’s findings align with previous research highlighting the urgent need for accessible HIV care and prevention information in U.S. Latino communities [11,37]. Latinos continue to experience high rates of new HIV infections, accounting for 33% of all new cases in 2022, and face significant barriers to care and limited awareness of prevention methods [2].

Addressing mental health in self-management interventions for LWH is crucial, as this population faces unique challenges such as higher levels of stigma, discrimination, and cultural barriers, which can worsen mental health issues like depression and anxiety [40]. These mental health challenges can negatively impact medication adherence and overall health outcomes [41]. Integrating culturally sensitive mental health support into self-management programs can improve HIV outcomes [42]. By prioritizing mental health, these interventions can help LWH develop coping strategies, improve quality of life, and achieve better health outcomes.

### Limitations

While the development and initial evaluation of *ViviendoPositivos* shows promise, we must acknowledge several limitations. First, the data collected in this study were self-reported, which may have led to the possibility of recall and social desirability bias. Second, the convenience sample in this study may not be representative of all the diversity within Latino subgroups from other locations outside Colorado. While the majority of participants in this study were male (88%), this reflects the demographics of LWH in Colorado, where men comprise a significant proportion of those affected [23]. However, we acknowledge that this gender distribution may limit the transferability of our findings to other subgroups, including women and non-binary individuals. Future studies should aim to include a more diverse sample to ensure the intervention’s broader relevance and effectiveness.

Third, the study focused primarily on the telenovela’s acceptability without an assessment of its impact on specific health outcomes (e.g., ART adherence, HIV-related knowledge, or quality of life). Although participants expressed a willingness to engage with the telenovela, it remains unclear whether this engagement translates into meaningful behavior change or improved health outcomes. A larger study is needed to rigorously evaluate the efficacy and effectiveness of *ViviendoPositivos*. Despite these limitations, this is the first attempt to develop a culturally tailored telenovela intervention to improve HIV self-management outcomes among LWH.

### Conclusions

This study described the process for developing a culturally tailored filmed telenovela, *ViviendoPositivos*, and evidence as an acceptable intervention to promote self-management among LWH. This intervention demonstrates the importance of developing culturally tailored interventions using a community engagement approach in promoting HIV care health behaviors and reducing risk behaviors. Study findings highlight the ongoing need for testing and implementing *ViviendoPositivos* among LWH to increase its scalability, accessibility, and potential for dissemination.

## Supporting information

S1 File Supplemental Table 1. Joint display for quantitative and qualitative data.
(DOCX)

S2 File Quantitative data. (CSV)
