# Supplementary material for: Development and acceptability of ViviendoPositivos: A culturally tailored telenovela (soap opera) intervention to improve self-management among Latinos with HIV
Source: PLoS One. 2025 Jun 30;20(6):e0326930. doi: 10.1371/journal.pone.0326930 (PMC12208486; doi:10.1371/journal.pone.0326930)
Supplement: S1 File — Joint display for quantitative and qualitative data. (DOCX) [file pone.0326930.s001.docx]

**Supplemental Table 1. Joint display for quantitative and qualitative data**

| **Construct** | **Qualitative** | **Quantitative** | **Convergence vs. divergence** | **Meta inferences** |
| --- | --- | --- | --- | --- |
| Satisfaction with the telenovela stories | Theme 1: Satisfaction  • *Format:* Engaging, well-produced, and natural delivery of HIV education.  • *Stories:* Realistic and relatable; encouraged interest in support groups.  • *Content:* Clear, detailed, and informative (e.g., U=U, CD4, PrEP). | • 92% satisfied with the information.  • 92% satisfied with the stories.  • 71% rated it "very good"; 29% "good". | Convergence: Strong alignment between positive qualitative feedback and high satisfaction ratings. | Participants expressed high satisfaction with the telenovela. The engaging format, relatable storylines, and accessible content were well-received and aligned with high satisfaction ratings, suggesting strong acceptance and engagement. |
| Perceived utility of the telenovela | Theme 2: Perceived utility  • *Holistic management of HIV:* Encouraged physical, mental, and social self-care.  • *HIV prevention education*: Provided accurate info, reduced stigma and misinformation. | • 88% said topics and issues were important to them. | Convergence: Qualitative insights support the quantitative perception of utility. | Participants viewed the telenovela as a valuable tool for both HIV self-management and prevention education. It addressed their needs and aligned with their lived experiences, enhancing relevance and perceived usefulness. |
| Suggestions to improve the telenovela stories | Theme 3: Modifications  • *Storyline:* Suggestions for character diversity, more humor, deeper personal storytelling. • *Content reinforcement:* Emphasize U=U, PrEP, condoms, and include takeaways. • *Audience inclusion:* More cultural, linguistic, and experiential inclusivity. | • 100% willing to watch the telenovela. • 96% would recommend it to others. | Partial convergence: Enthusiasm was high, but qualitative data highlighted areas for improvement not captured in quantitative metrics. | Despite strong interest and endorsement, participants offered thoughtful suggestions to improve representation, content clarity, and inclusivity, indicating areas for refinement and increased impact in future iterations. |
